# Soil organic matter widens the range of water contents for tillage

**DOI:** 10.1016/j.still.2018.05.001

**Published:** 2018-10

**Authors:** Peter Bilson Obour, Johannes L. Jensen, Mathieu Lamandé, Christopher W. Watts, Lars J. Munkholm

**Affiliations:** a Department of Agroecology, Aarhus University, Research Centre Foulum, Blichers Allé 20, P.O. Box 50, DK-8830, Tjele, Denmark; b Faculty of Environmental Sciences and Natural Resource Management, Norwegian University of Life Sciences, P.O. Box 5003 NMBU, 1432, Ås, Norway; c Department of Sustainable Agriculture Sciences, Rothamsted Research, Harpenden, United Kingdom

**Keywords:** Consistency approach, Soil organic carbon, Water retention approach

## Abstract

The effects of soil organic matter on the water contents for tillage were investigated by sampling soils with a uniform texture, but a range of soil organic carbon (SOC) from two long-term field experiments at Highfield in Rothamsted Research, UK and Askov Experimental Station, Denmark. The treatments studied in Highfield were Bare fallow (BF), Continuous arable rotation (A), Ley-arable (LA) and Grass (G); and in Askov: unfertilized (UNF), ½ mineral fertilizer (½ NPK), 1 mineral fertilizer (1NPK), and 1½ animal manure (1½AM). Minimally disturbed soil cores (100 cm^3^) were sampled per plot in both locations from 6 to 10 cm depth to generate water retention data. Soil blocks were also sampled at 6–15 cm depth to determine basic soil properties and to measure soil aggregate strength parameters. The range of soil water contents appropriate for tillage were determined using the water retention and the consistency approaches. SOC content in Highfield was in the order: G > LA = A > BF, and in Askov: 1½ AM > 1NPK = ½NPK > UNF. Results showed that different long-term management of the silt loam Highfield soil, and fertilization of the sandy loam Askov soil affected the mechanical properties of the soils— for Highfield soil, aggregates from the G treatment were stronger in terms of rupture energy when wet (−100 hPa matric potential) than the BF treatment. As the soil dried (−300 and −1000 hPa matric potentials), soil aggregates from the G treatment were relatively weaker and more elastic than the BF soil. Our study showed, for both Highfield and Askov soils, a strong positive linear increase in the range of water contents for tillage with increasing contents of SOC. This suggests that management practices leading to increased SOC can improve soil workability by increasing the range of water contents for tillage. We recommended using the consistency approach over the water retention approach for determining the range of water contents for tillage because it seems to give realistic estimates of the water contents for tillage.

## Highlights

• Soil organic carbon (SOC) affected the mechanical properties of soil aggregates.
• Water contents for tillage is determined using water retention and consistency approaches.
• There is a strong positive relation between SOC and range of water contents for tillage (Δ*θ*_RANGE_).
• Δ*θ*_RANGE_ determined based on the consistency approach is recommended over the water retention approach.

## 1 Introduction

Tillage plays an important role in arable farming. One of the primary purposes of tillage is for seedbed preparation, where operations are designed to alter soil bulk density, aggregate size distribution and other soil physical characteristics to create soil conditions and environment favoring crop establishment, germination and growth (Johnsen and Buchle, 1969).

Tillage can be performed over a range of water content (Δ*θ*_RANGE_) where soil is workable. In this study, soil workability is defined as the ease of working with a well-drained soil to produce desirable seedbeds (Dexter, 1988), i.e. not consisting of fragments that are either too fine or too coarse for crop establishment. Δ*θ*_RANGE_ is the difference between the wet tillage limit (*θ*_WTL_) and the dry tillage limit (*θ*_DTL_). *θ*_WTL_ and *θ*_DTL_ are the upper and lower water contents for tillage, respectively. Optimum water content for tillage (*θ*_OPT_) is the water content where tillage produces maximum number of smaller fragments and minimum number of large fragments (clods) (Dexter and Bird, 2001). Russell (1961) suggests that small soil fragments that create ideal seedbeds as those consisting 1–5 mm in size. The water contents for tillage have been estimated using the water retention approach (e.g., Dexter and Bird, 2001) and the consistency approach (e.g., Munkholm et al., 2002).

Performing tillage when soil is too wet can lead to structural damage due to remolding and puddling (Dexter and Bird, 2001). Likewise, executing tillage when soil is too dry requires high specific energy because soil is strong (Hadas and Wolf, 1983). Therefore, knowledge of *θ*_WTL_ and *θ*_DTL_ and the effects of soil physical properties on these limits are crucial. Such knowledge can provide practical information on the satisfactory Δ*θ*_RANGE_ over which tillage operations produce desirable soil structures for crop establishment and growth (Obour et al., 2017). Further, knowledge of the suitable water contents for tillage can be used in a decision support system to reduce the risk of structural damage, and the use of excessive energy during tillage (Sørensen et al., 2014).

Soil organic carbon content (SOC) is a critical soil property that affects many other soil physical properties and functions. Organic binding agents such as roots and fungal hyphae play an important role in soil aggregation and stabilization (Tisdall and Oades, 1982), and improves soil resistance and resilience to external stresses (Gregory et al., 2009). SOC also affects soil mechanical properties such as soil strength, bulk density, inter-aggregate or structural porosity, and enhances better soil fragmentation during tillage (Abdollahi et al., 2014). It also influences infiltration, drainage and water storage — it improves water retention due to high absorptive capacity for water (Murphy, 2015), and increases soil strength in wet conditions, which increases *θ*_WTL_. In soils with small content of SOC, clay dispersion is higher (Watts and Dexter, 1997; Jensen et al., 2017), which may increase soil strength due to crusting and cementation on drying, consequently affecting the *θ*_DTL_. There are few studies that have investigated the effect of SOC on the water contents for tillage. Although Dexter and Bird (2001) investigated the water contents for tillage for a silt loam in Highfield using the water retention approach, and Munkholm et al. (2002) a sandy loam soil in Askov using the consistency approach, they did not evaluate this effect statistically. There remains a need for more quantitative information on the SOC/water content relationship and its influence on tillage (Obour et al., 2017). Such information will help improve knowledge on how the physical condition of soil for tillage changes with changing SOC. In the present study, we investigated the effect of SOC on the water contents for tillage using both the water retention and consistency approaches to expand the findings of the previous studies. Our study focuses on water contents for secondary tillage used for seedbed preparation. It relates to unconfined fragmentation of soil aggregates rather than shearing of bulk soil.

The objectives of this study were to: (i) quantify the effect of SOC on the mechanical behavior of soil aggregates and the water contents for tillage, and (ii) evaluate the water retention and consistency approaches for determining the range of water contents for tillage. We hypothesized that the range of water contents for tillage increases with increasing SOC content.

## 2 Materials and methods

### 2.1 The experiments

Soil samples were taken from two long-term field experiments; the Highfield long-term, ley/arable experiment at Rothamsted Research, UK (51°80′N, 00°36′W) and from the Askov long-term experiment on animal manure and mineral fertilizers at Askov Experimental Station, Denmark (55° 28ʹ N, 09°07ʹE). These soils had uniform textures, but a range of SOC.

The soil from Highfield is a silt loam classified as Chromic Luvisol according to the World Reference Base (WRB) soil classification system (Watts and Dexter, 1997). The experimental site was originally established with grass, but for ∼56 years prior to sampling, each of the plots has an unbroken history under its present management. As a consequence, the soil has a wide SOC gradient in the topsoil along the Bare fallow (BF), Continuous arable rotation (A), Ley-arable (LA) and Grass (G) treatments in the order: G > LA = A > BF (Table 1). The G treatment has been known as Reseeded grass, but throughout this paper, it will be called ‘Grass (G)’ treatment. The A, LA and G treatments were included in a randomized block design with four field replicates, whereas the four BF replicates were not part of the original design and were located at one end of the experimental site.

**Table 1:** Basic soil properties and water retention characteristics of the two soils investigated.

	Highfield soil^a^				Askov soil^b^			
	BF	A	LA	G	UNF	½NPK	1NPK	1½AM
SOC (g 100 g^−1^ minerals)	0.90	1.73a*	2.16a*	3.29b*	0.95a	1.07b	1.13b	1.33c
Clay <2 μm (g 100 g^−1^ minerals)	27	26	26	26	9	10	10	10
Fine silt 2–20 μm (g 100 g^−1^ minerals)	25	26	26	27	9	10	9	10
Coarse silt 20–63 μm (g 100 g^−1^ minerals)	33	32	32	32	16	16	17	16
Sand 63–2000 μm (g 100 g^−1^ minerals)	15	16	16	15	65	64	64	65
Bulk density (g cm^−3^)	1.45	1.39b	1.21a*	1.13a*	1.54a	1.51a	1.41b	1.42b
Pores <30 μm (m^3^ m^−3^)	0.31	0.39a	0.39a	0.46b	0.21	0.23	0.22	0.25
Pores >30 μm (m^3^ m^−3^)	0.15	0.09a	0.15b	0.10a	0.19a	0.19a	0.24b	0.21ab
*θ*_PL_ (kg kg^−1^ oven dried soil)^c^	0.19	0.24a*	0.25a*	0.34b*	0.15	0.17	0.17	0.18

Treatments labelled with different letters in a given row for each soil are significantly different. Pairwise comparison for differences between Arable (A), Ley-arable (LA) and Grass (G) treatments at Highfield and between unfertilized (UNF), ½ mineral fertilizer (½NPK), 1 mineral fertilizer (1NPK), and 1½ animal manure (1½AM) treatments at Askov. Paired *t*-test for differences between Bare fallow (BF) and A, LA and G at *p* < 0.05. Values of A, LA and G with an asterisk (*) indicate it is significantly different from BF treatment based on the paired *t*-test.

*θ*_PL_: water content at plastic limit.

a Data from Jensen et al. (2018).

b Data from Jensen et al. (2017).

c Data not reported in Jensen et al. (2017) and Jensen et al. (2018).

The soil from the Askov experimental site is a sandy loam classified as an Aric Haplic Luvisol according to the WRB classification system (IUSS Working Group WRB, 2015). The experiment includes the following four nutrient treatments: Unfertilized plots (UNF), and plots that have received ½ mineral fertilizer (½NPK), 1 mineral fertilizer (1NPK), and 1½ animal manure (1½AM). The nutrient treatments represent ½, 1 and 1½ times the standard rate of a given crop for total nitrogen (N), phosphorus (P), and potassium (K) in AM or NPK fertilizer (Christensen et al., 2017). The experiment utilizes a randomized block design with three field replicates. The different levels of nutrients applied results in a SOC gradient among the treatments in the order: 1½AM > 1NPK = ½NPK > UNF plots (Table 1). Crop management has been a four-course rotation of winter wheat (*Triticum aestivum* L.), silage maize (*Zea mays* L.), spring barley (*Hordeum vulgare* L.), and a grass-clover mixture (*Trifolium hybridum* L.*, Medicago sativa* L.*, Lotus corniculatus* L.*, Lolium perenne* L.*, Festuca pratensis* Huds and *Phleum pratense* L.) used for cutting in the following year (Jensen et al., 2017).

Table 1 shows the basic characteristics of the studied soils. For a more detailed description of the experiment and treatments in Askov and in Highfield reference is made to Jensen et al. (2017) and Jensen et al. (2018), respectively. From here on the soils are referred to with the treatment labels explained above.

### 2.2 Sampling

At Askov, sampling took place in September 2014 following a winter wheat crop. At Highfield, sampling was done in March 2015. At both Askov and Highfield, soil cores (6.1 cm diameter, 3.4 cm high, 100 cm^3^) were taken from 6 to 10 cm depth by inserting steel cylinders gently into the soil. Six soil cores were sampled per plot at both locations. In addition, soil blocks were sampled at 6–15 cm depth: Two soil blocks (4000 cm^3^) per plot in Askov, and three blocks (2750 cm^3^) per plot in Highfield. The soil cores were stored in a field moist condition in a 2 °C room until analysis. Portions of the soil blocks per plot were spread out on a table and carefully fragmented by hand along natural planes of weakness and left to dry in a ventilated room ∼20 °C.

### 2.3 Basic chemical and physical analysis

Air-dry soil samples from each plot was crushed to <2 mm and SOC was determined by dry combustion using Flash 2000 NC Soil Analyzer (Thermo Fisher Scientific, Waltham, MA, USA). Soil texture was determined on portions of the <2 mm samples using a combined hydrometer/sieving method after removal of soil organic matter by hydrogen peroxide (Gee and Or, 2002).

### 2.4 Soil water retention

To obtain water retention curves, water content was measured from the six soil cores per plot from Askov at −10, −30, −100 and −300 hPa matric potentials; and at −10, −30, −100, −300 and −1000 hPa matric potentials for Highfield soil on tension tables, vacuum pots and pressure plates (Dane and Hopmans et al., 2002). Water content at −15,000 hPa matric potential was determined from air-dry <2 mm samples using WP4-T Dewpoint Potentiometer (Scanlon et al., 2002). Following equilibrium at each water potential the soil cores were oven dried at 105 °C for 24 h. Soil bulk density of each soil core was calculated from the mass of the oven-dried soil divided by the total soil volume. Bulk density was corrected for stone weight and volume for Highfield soil samples because they contained a significant amount of stones. Porosity was estimated from bulk density and particle density, where particle density was measured on one plot from each treatment using the pycnometer method (Flint and Flint, 2002). For the remaining plots, the particle density was predicted from SOC by a linear regression model. The pore size distributions of the soils were estimated from the water retention measurements, assuming the approximate relation:

(1) d=−3000/Ψ

where *d* is equivalent cylindrical pore diameter (μm) and *ψ* is the soil matric potential (hPa).

### 2.5 Plastic limit

Plastic limit (PL) was determined using the standard ASTM (Casagrande) test procedure (McBride, 2007). In brief, for each plot, about 15 g of air-dry soil was sieved to <1 mm and then mixed with water until it became plastic and easily molded into a ball. About 8 g of the soil was rolled between the fingers and a smooth glass plate. PL was determined as the gravimetric water content where the soil began to crumble when rolled into a thread of approximately 3.2 mm in diameter (McBride, 2007).

### 2.6 Calculations of water contents for tillage

The water contents for tillage were determined using two approaches: (i) water retention approach, and (ii) consistency approach.

#### 2.6.1 Water retention approach

Dexter and Bird (2001) and Dexter et al. (2005) suggested that the water contents for tillage can be estimated from the parameters of the soil water retention curve using the van Genuchten (1980) water retention equation.

The gravimetric water content (*θ,* kg kg^−1^) corresponding to each matric potential (hPa) was calculated by fitting the van Genuchten equation with the Mualem (1976) restriction of *m = *1-1*/n* to each set of water retention data obtained from each plot at Askov and Highfield:

(2) $$\theta \;=\;(\theta_{SAT}\;-\;\theta_{RES})\;{\left[1\;+\;{\left(\alpha h\right)}^{n}\right]}^{1\;-\;\left(1/n\right)}\;+\;\theta_{RES}$$

where *θs_AT_* and *θ_RES_* are the water contents at saturation, i.e. at h* = 0*, and the residual water contents, h* = ∞,* respectively, *α* is a scaling factor for h; and *n* is a fitted parameter that controls the shape of the curve. *θ_RES_* was set equal to zero. Values of *n* were obtained using the curve-fitting program, RETC (van Genuchten et al., 1991).

The wet tillage limit (*θ*_WTL_) was estimated as follows:

(3) $$\theta_{WTL}\;=\;\theta_{INFL}\;+\;0.4\;\left(\theta_{SAT}\;-\;\theta_{INFL}\right)$$

The optimum water content for tillage (*θ*_OPT_) was estimated as water content at the inflection point of the soil water retention curve (*θ*_INFL_):

(4) $$\theta_{INFL}=\theta_{SAT}{\left[1+\frac{1}{1-\left(1/n\right)}\right]}^{1-\left(1/n\right)}$$

The matric potential at the dry tillage limit (h_DTL_) was estimated as proposed by Dexter et al. (2005):

(5) $$h_{DTL}\approx \frac{2}{\alpha }{\left[\frac{1}{1-\left(1/n\right)}\right]}^{1/n}n^{1.1}$$

The corresponding water content at the dry tillage limit (*θ*_DTL_) was calculated by putting the value of h_DTL_ from Eq. (5) into (2) yielding:

(6) $$\theta_{DTL}=\theta_{SAT}{\left[1+{\left(\alpha h_{DTL}\right)}^{n}\right]}^{1-\left(1/n\right)}$$

The range of water contents for tillage using the water retention approach (Δ*θ*_RANGE_ (water retention)) was calculated as:

(7) Δ*θ_RANGE_* (water retention) = *θ_WTL_*–*θ_DTL_*

#### 2.6.2 Consistency approach

The water contents for tillage based on the consistency approach were determined as follows:

*θ*_WTL_ and *θ*_OPT_ were determined according to Dexter and Bird (2001):

(8) *θ_WTL_ = θ_PL_*

(9) *θ_OPT_* = 0.9 *θ_PL_*

*θ*_DTL_ was graphically determined for each plot as water content at twice the strength at *θ*_OPT_ from the relation between natural logarithm of tensile strength (*Y)* of 8–16 mm soil aggregates and gravimetric water content measured at different matric potentials (Munkholm et al., 2002). Examples of how it was determined are shown in Section 3.5.

The range of water contents for tillage using the consistency approach (Δ*θ*_RANGE_ (consistency)) was calculated as described by Munkholm et al. (2002):

(10) Δ*θ_RANGE_* (consistency) = *θ_WTL_*–*θ_DTL_*

### 2.7 Aggregate tensile strength

#### 2.7.1 Highfield soil

We crushed portions of the air-dry soil using the rolling method suggested by Hartge (1971). The crushed soil was passed through a nest of sieves with 8–16, 4–8, 2–4 and 1–2 mm of apertures to obtain four different aggregate size fractions. Some of the 8–16 mm air-dry aggregates were selected randomly from each sampling plot, saturated by capillarity and then drained to −100, −300 and −1000 hPa matric potentials using tension tables, vacuum pots and pressure plates, respectively. Fifteen aggregates were selected at random from each size fraction of the air-dry aggregates (8–16, 4–8, 2–4 and 1–2 mm), and the 8–16 mm aggregates equilibrated at the three matric potentials. These aggregates were used to measure *Y* using the indirect tension test (Rogowski, 1964). This test assumes brittle fracture theory and we checked we did not exceed the 20% maximum strain limit for onset of plastic deformation (Kuhn and Medlin, 2000); particularly when aggregates were tested at a wetter state (−100 hPa matric potential). Each of the aggregates was weighed individually and subjected to indirect tension testing by crushing the individual aggregates between two parallel plates (Rogowski, 1964) using an automatically operated mechanical press (Instron Model 5969, Instron, MA,USA). The point of failure for each aggregate was automatically detected when a continuous crack or sudden drop in force (40% of the maximum load) was read. The maximum force at failure was automatically recorded by a computer program. After the test, the crushed aggregates were oven-dried at 105 °C for 24 h to determine their gravimetric water content.

#### 2.7.2 Askov soil

Portions of the field-moist soil was fragmented by hand and sieved to obtain 8–16 mm aggregates. These aggregates were divided into three groups based on their moisture status: air-dry, air-dry rewetted to field capacity (−100 hPa matric potential (Munkholm and Kay, 2002)) and field moist aggregates. Aggregate tensile strength for Askov soil was measured as described in Jensen et al. (2017).

For both Highfield and Askov soils, *Y* was calculated from the equation suggested by Dexter and Kroesbergen (1985):

(11) *Y* = 0.567*F*/*d^2^*

where 0.576 is the proportionality constant resulting from the relation between the compressive load applied and the tensile stress exerted on the aggregate. *F* is the maximum force (N) at failure and *d* is the effective diameter of the spherical aggregate (m); it was obtained by adjusting the aggregate diameter according to the individual masses (Dexter and Kroesbergen, 1985):

(12) *d = d_1_(m_0_/m_1_)^1/3^*

where *d_1_* = is the diameter of aggregates defined by the average sieve sizes (e.g., 0.012 m for 8–16 mm aggregates), *m_0_* is the mass (g) of the individual aggregate and *m_1_* is the mean mass of a batch of aggregates of the same size class (in this case 15 aggregates for each size fractions).

Rupture energy (*E_r_*) was calculated from the area under the stress-strain curve up the point of tensile failure (Vomocil and Chancellor, 1969):

(13) *E_r_≈Σ_i_ F(s_i_)*Δ*s_i_*

where *F(s_i_)* denotes the mean force at the *i*th subinterval and Δ*s_i_ si* the displacement length of the *i*th subinterval. The mass specific rupture energy (*E_sp_*) was defined on gravimetric basis from the equation:

(14) *E_sp_*  = *E_r_/m*

where *m* is the mass of the individual aggregates.

Young's modulus (*E*) was determined to obtain a quantitative measure of stiffness (elasticity) of the aggregates (determined only for the Highfield samples). It was estimated from the gradient of the stress-strain curve to the elastic limit, assuming linearity up to that point, which was determined using a macro program:

(15) *E = σ / ԑ*

where *σ* is stress (Pa) and *ԑ* is strain.

### 2.8 Statistical analysis

All statistical analyses were carried out in R software package (R Core Team, 2017). The *Y, E_sp_* and *E* data were log-transformed (ln) to yield normal distribution. The Highfield data were fitted to a linear mixed effect model, which comprised treatment as fixed and block as random factors. The Kenward-Roger method was used to calculate degrees of freedom. For the Askov data, treatment effects were analyzed using a linear model which comprised block as a fixed effect. We used *p* < 0.05 as a criterion for statistical significance of treatment effects. Where effect of treatment was found to be significant, further analyses were made to identify which treatment means were different (pairwise comparison) using the general linear hypotheses (*glht*) function implemented in R multcomp package. For the four BF replicates which were not included in the original randomized block design, a paired *t*-test was used to investigate if the treatment significantly differed from the A, LA and G treatments. We acknowledged that the paired *t*-test statistics performed to compare statistical significance difference between the BF treatment and the A, LA and G treatment was a less robust test. Throughout the presentation of Results (Section 3), statistical significant differences between the A, LA and G treatments based on the pairwise comparison are labeled with different letters, whereas statistical significant differences between the BF treatment compared to the A, LA and G treatments based on the paired *t*-test are shown by an asterisk (*) symbol against the A, LA or G treatment.

## 3 Results

### 3.1 Basic properties of the investigated soils

Soil bulk density was significantly greater for the BF and A soils than the LA and G treatments, and for the UNF and ½NPK compared to the 1NPK and 1½AM treatments (Table 1). There were more large pores >30 μm in the LA treatment compared to the G and A treatments from Highfield, and for the 1NPK than the UNF and ½NPK soils. Pores <30 μm, generally, increased with SOC. *θ*_PL_ was lower for the BF treatment than the other treatments at Highfield (Table 1). *θ*_PL_ increased with an increase in SOC at Highfield (R^2^ = 0.82, *p* < 0.001). The same was also seen at Askov, although not significant (R^2^ = 0.15, *p* = 0.21).

### 3.2 Tensile strength parameters of air-dry aggregates

In this section and in Sections 3.3 and 3.4, only results from Highfield are presented. Tensile strength parameters of the Askov soil have previously been reported in another study by Jensen et al. (2017). *Y* and *E_sp_* values for all the aggregate size fractions measured did not differ between the treatments (Table 2). Geometric mean of *E_sp_* value of all size fractions was greater for the G treatment (19.1 J kg^−1^) compared to the A and BF treatments (15.4 and 14.9 J kg^−1^, respectively). Aggregates for the size fraction 2–4 mm were more elastic for the G treatment than the A and LA treatments, whereas for 4–8 mm size fraction, the LA treatment was more elastic compared to both the A and G treatments. Geometric mean values of all size fractions showed that the G and LA treatments had lower *E* (high elasticity) compared to the BF treatment (Table 2).

**Table 2:** Geometric means of tensile strength (*Y*), mass specific rupture energy (*E_sp_*) and estimated Young’s modulus (*E*) of air-dry soil aggregates.

Soil attribute	Aggregate size	BF	A	LA	G
*Y* (kPa)	1–2 mm	617	544	637	526
	2–4 mm	534	570	530	492
	4–8 mm	394	365	361	307
	8–16 mm	419	400	363	279
	Mean	483	462	459	386

*E_sp_* (J kg^−1^)	1–2 mm	15.4	19.8	23.5	24.1
	2–4 mm	16.3	21.8	18.8	24.6
	4–8 mm	18.5	12	16.8	17.1
	8–16 mm	9.4	10.8	11.7	13.2
	Mean	14.9	15.4a	17.1ab	19.1b*

*E* (MPa)	1–2 mm	15.9	14.4	13.8	15.4
	2–4 mm	34.3	32.9b	32.6b	25.9a
	4–8 mm	36.1	44.5c	24.7a	34.7b
	8–16 mm	31.9	23.2	22.8	14.8
	Mean	28.2	26.4	22.4*	21.2*

Geometric means of all size fraction for *Y*, *E_sp_* and *E* are shown. Treatments labelled with different letters in a given row are significantly different. Pairwise comparison for differences between Arable (A), Ley-arable (LA) and Grass (G), and paired *t*-test for differences between Bare fallow (BF) and A, LA and G at *p* < 0.05. Values of A, LA and G with an asterisk (*) indicate it is significantly different from BF treatment based on the paired *t*-test.

### 3.3 Tensile strength parameters of rewetted aggregates

As expected, for all treatments, *Y*, *E_sp_* and *E* all increased as the soil dries: the soils become stronger and stiffer. At wet and wet–moist state (−100 and −300 hPa matric potentials), *Y* values did not differ significantly between treatments, whereas at moist–dry state (−1000 hPa matric potential), aggregates for the LA and G soils had lower *Y* compared to the A treatment (Table 3). Conversely, the G soil with large SOC had higher *E_sp_* at −100 hPa matric potential than the other treatments. On the other hand *E_sp_* was not significantly different between treatments when aggregates were tested at −300 and −1000 hPa matric potentials (Table 3). Similar to the air-dry aggregates, lower *E* was observed for the G aggregates at −300 and −1000 hPa matric potentials compared to the BF treatment (Table 3).

**Table 3:** Geometric mean of tensile strength (*Y*), mass specific rupture energy (*E_sp_*) and estimated Young’s modulus (*E*) of 8–16 mm soil aggregates adjusted at −100, −300 and −1000 hPa matric potentials.

Matric potential	Soil attribute	BF	A	LA	G
−100 hPa	*Y* (kPa)	14.6	15.3	15.2	15.8
	*E_sp_* (J kg^−1^)	0.55	0.62a	0.86a	1.64b*
	*E* (MPa)	0.83	0.83b	0.73a	0.68a

−300 hPa	*Y* (kPa)	23.0	27.3	23.5	20.1
	*E_sp_* (J kg^−1^)	1.04	1.36	1.31	1.68
	*E* (MPa)	1.20	1.00	0.87*	0.82*

−1000 hPa	*Y* (kPa)	38.5	45.1b	30.7a	25.9a*
	*E_sp_* (J kg^−1^)	1.49	2.05	1.50	2.15
	*E* (MPa)	2.43	1.81c	1.42b*	1.09a*

Treatments labelled with different letters in a given row are significantly different. Pairwise comparison for differences between Arable (A), Ley-arable (LA) and Grass (G), and paired *t*-test for differences between Bare fallow (BF) and A, LA and G at *p* < 0.05. Values of A, LA, and G with an asterisk (*) indicate it is significantly different from BF treatment based on the paired *t*-test.

### 3.4 Relationship between strength parameters of air-dry aggregates and soil organic carbon

**Fig. 1:**
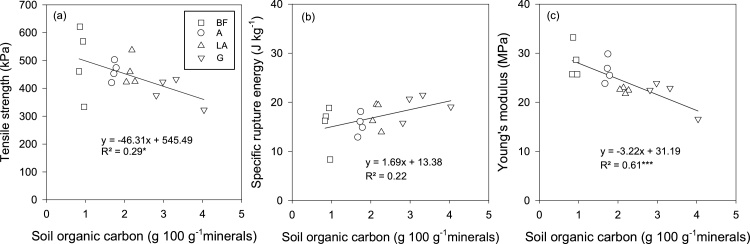
(a) Tensile strength, (b) Mass specific rupture energy and (c) Young’s modulus of air-dry aggregates calculated as geometric means across the four aggregate classes (8–16, 4–8, 2–4 and 1–2 mm) for each plot as a function of soil organic carbon. Bare fallow (BF), Arable (A), Ley-arable (LA) and Grass (G) treatments, and Unfertilized (UNF), ½ mineral fertilizer (½NPK), 1 mineral fertilizer (1NPK), and 1½ animal manure (1½AM) treatments. *p < 0.05 and ***p < 0.001.

Geometric mean of *Y*, *E_sp_* and *E* across the four aggregate size fractions (8–16, 4–8, 2–4 and 1–2 mm) were related to SOC content. There was a negative linear decrease in *Y* with increasing SOC content (*p* < 0.05). A stronger negative linear relationship was found between SOC and *E* (*p* < 0.001). In contrast, there was a positive linear increase in *E_sp_* with increasing SOC content, although not significant (*p* = 0.07) (Fig. 1a–c). Overall, 29%, 22% and 61% of the variation in *Y*, *E_sp_*, and *E*, respectively of aggregates could be explained by SOC (Fig. 1a–c).

### 3.5 Water contents for tillage

**Fig. 2:**
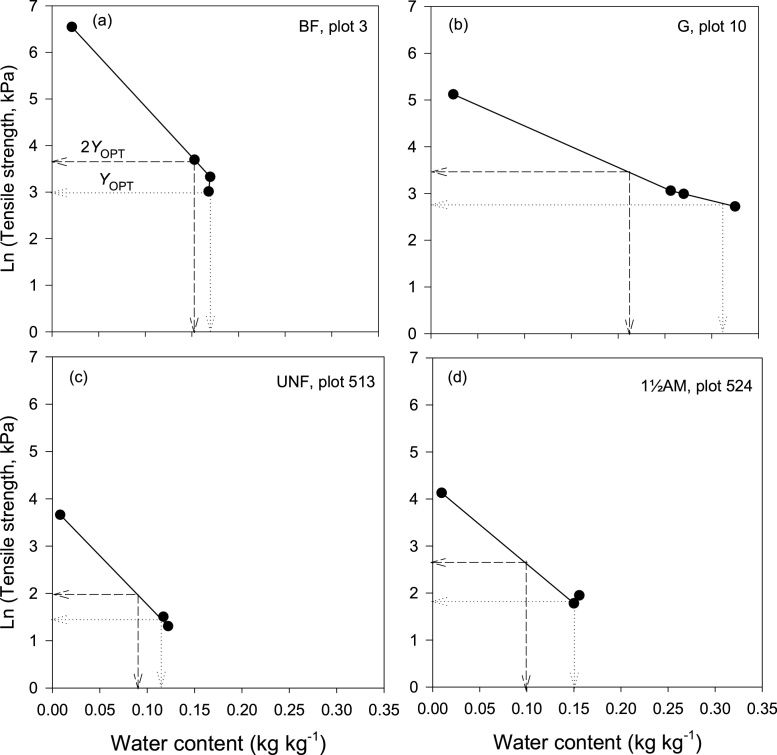
Graphical approach for determining *θ*_DTL_: For Highfield, from natural logarithm of tensile strength of 8–16 mm soil aggregates related to gravimetric water content determined on the aggregates at −100, −300, −1000 hPa matric potentials and at air-dry state for (a) Bare fallow (BF) soil and (b) Grass (G) soil. For Askov, from natural logarithm of tensile strength of 8–16 mm aggregates related to gravimetric water content determined on the aggregates at field capacity, field moist and air-dry state for (c) Unfertilized (UNF) soil and (d) 1½ animal manure (1½AM) soil (n = 4 for Highfield, n = 3 for Askov).

Water content at dry tillage limit (*θ*_DTL_) for each plot was graphically determined from the relationship between *Y* of aggregates in the 8–16 mm size range and the gravimetric water content at −100, −300, −1000 hPa matric potentials and at air-dry state. Examples of how we determined water content at twice the strength at *θ*_OPT_ for the BF and G soils from Highfield, and the UNF and 1½AM soils from Askov are presented in Fig. 2a– d. For these examples, water content at *θ*_DTL_ for the BF soil was 0.16 kg kg^−1^ and 0.22 kg kg^−1^ for the G soil. *θ*_DTL_ for the UNF and 1½AM soil were 0.09 and 0.10 kg kg^−1^, respectively.

**Fig. 3:**
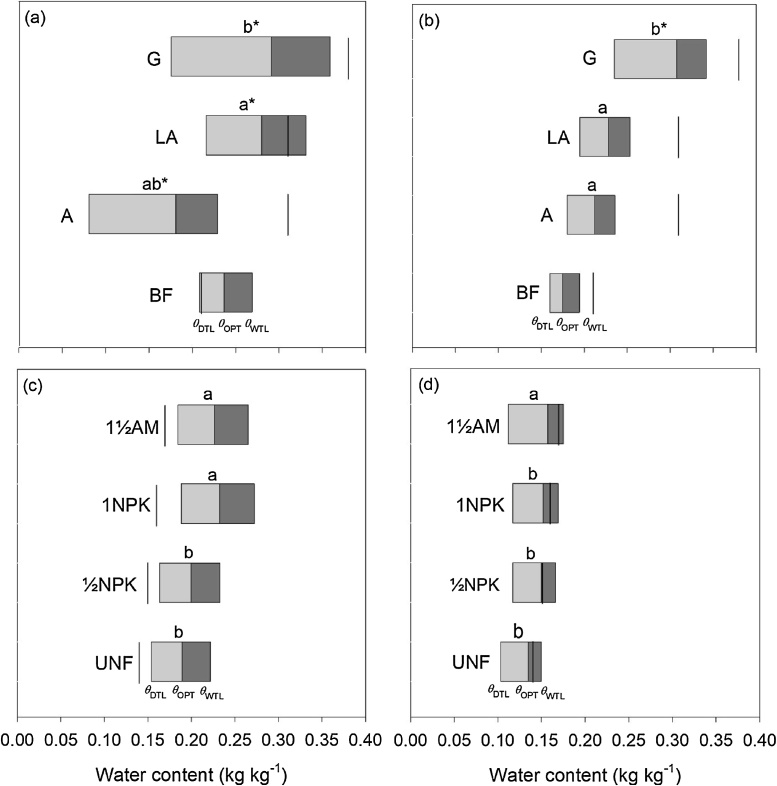
Water contents for tillage based on the water retention approach (a and c), and the consistency approach (b and d) for Highfield and Askov soils. *θ*_DTL_ (dry tillage limit), *θ*_OPT_ (optimum water content for tillage) and *θ*_WTL_ (wet tillage limit). Solid short vertical lines show water content at −100 hPa matric potential. For Highfield soils, treatments labelled with different letters are significantly different. Pairwise comparison for differences between Arable (A), Ley-arable (LA) and Grass (G), and paired *t*-test for differences between Bare fallow (BF) and A, LA and G at *p* < 0.05. Values of A, LA, and G with an asterisk (*) indicate it is significantly different from BF treatment based on the paired *t*-test. At Askov: Unfertilized (UNF), ½ mineral fertilizer (½NPK), 1 mineral fertilizer (1NPK), and 1½ animal manure (1½AM) treatments. Treatments with different letters are significantly different (*p* < 0.05).

The Δ*θ*_RANGE_ (water retention) and Δ*θ*_RANGE_ (consistency) are presented in Fig. 3a and b for Highfield soil, and Fig. 3c and d for Askov soil. *θ*_DTL_, *θ*_OPT_, *θ*_WTL_ at treatment levels are also shown for the two approaches. The G treatment with high SOC content had wider Δ*θ*_RANGE_ compared to the BF treatment at Highfield; and for the 1½AM compared to the UNF at Askov. Based on the water retention approach, Δ*θ*_RANGE_ for the G and BF treatments were 0.18 and 0.06 kg kg^−1^, respectively (Fig. 3a), and 0.08 and 0.07 kg kg^−1^ for the 1½AM and UNF treatments (Fig. 3c). Similar trends were seen for the consistency approach indicating that Δ*θ*_RANGE_ (consistency) for the G treatment was 0.11 kg kg^−1^ compared to 0.03 kg kg^−1^ for the BF treatment, and 0.06 kg kg^−1^ for the ½AM treatment compared to 0.05 kg kg^−1^ for the UNF treatment (Fig. 3b and d).

**Fig. 4:**
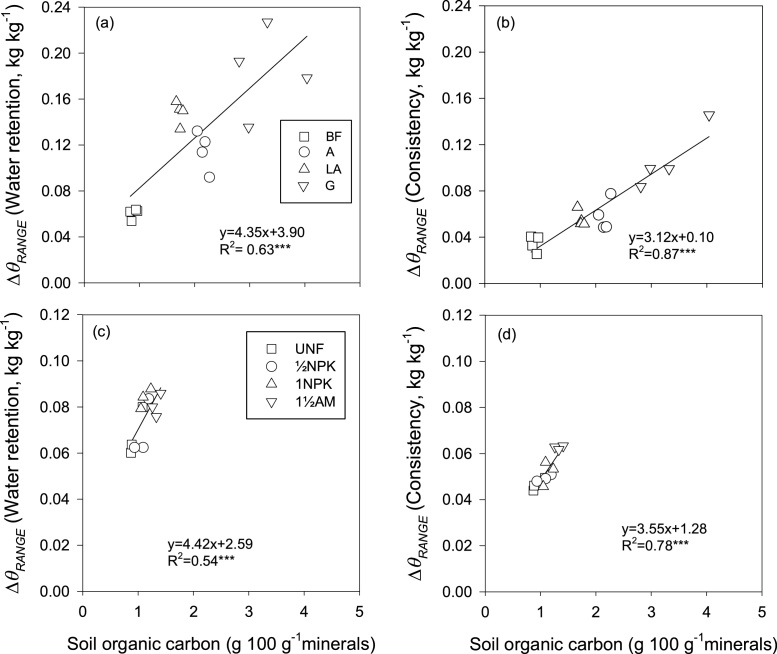
Δ*θ*_RANGE_ (water retention) and Δ*θ*_RANGE_ (consistency) as a function of soil organic carbon content for the Highfield (4a and b) and the Askov (4c and d) soils. Bare fallow (BF), Arable (A), Ley-arable (LA) and Grass (G) treatments, and Unfertilized (UNF), ½ mineral fertilizer (½NPK), 1 mineral fertilizer (1NPK), and 1½ animal manure (1½AM) treatments. Lines indicate linear regression. ***p < 0.001.

SOC content had a highly significant positive effect on Δ*θ*_RANGE_ (Fig. 4a–d). The effect of SOC content on Δ*θ*_RANGE_ (consistency) was more significant and more of the variation was explained (Fig. 4b and d) than with Δ*θ*_RANGE_ (water retention) (Fig. 4a and c).

## 4 Discussion

### 4.1 Effect of soil organic carbon content on aggregate strength parameters

The indirect tension test causes soil aggregates (or cores) to fail along pre-existing failure zones, and planes of weakness making *Y* a potentially sensitive measure of soil structural condition. Results showed that SOC had a negatively and a significant effect on geometric mean of *Y* across the four aggregate size classes when air-dry (Fig. 1a). This can be interpreted as *Y* reflects the degree of aggregation in a soil; it is influenced by aggregate porosity and bonds, failure planes within the aggregates and abundance of internal micro-cracks within the aggregates, which in turn are influenced by SOC (Watts and Dexter, 1998; Blanco-Canqui and Lal et al., 2006). Studies investigating the effect of SOC on aggregate strength show that for soil with less SOC, *Y* decreases with increasing soil moisture content whereas for soil with large SOC, aggregates are relatively stronger when wet and weaker when dry. For examples, Causarano (1993) and Munkholm et al. (2002) found that for clay and sandy loam soils, respectively with large SOC content, aggregates were stronger at water content at field capacity and weaker when air-dry. This may imply that wet soils do not slump under their own weight when wet during the winter and are relatively weak when dry; leading to easier root penetration and tillage. For the silt loam soil investigated here, *Y* did not significantly differ between the treatments at −100 and −300 hPa matric potentials (Table 3). However, when tested at −1000 hPa, *Y* was lower for the G treatment, 25.9 kPa compared to the BF and A treatments, 38.5 and 45.1 kPa, respectively (Table 3). Our results are consistent with Jensen et al. (2017) who found no significant difference in *Y* between the 1½ AM with large SOC content and the UNF treatment with small SOC content for aggregates at field capacity (−100 hPa matric potential) for the sandy loam soil at Askov. Results here suggest that the range of water content for measurement of *Y* is important to study the effect of SOC on soil aggregate strength.

Perfect and Kay (1994) suggested using rupture energy for the statistical characterisation of aggregates in tillage studies. They argued that, unlike *Y*, *E_sp_* does not involve any assumption of the mode of failure, making it more appropriate for estimating the strength of dry aggregates. Munkholm and Kay (2002) highlighted that *E_sp_* is also appropriate for estimating the strength and fragmentation of wet aggregates. We observed that at −100 hPa matric potential, *E_sp_* was significantly greater for the G compared to the other treatments at Highfield. This could be ascribed to the influence of SOC including organic binding and bonding materials such as polysaccharides fungal hyphaes and roots (Tisdall and Oades, 1982). Previous study of the BF, A and G treatments showed more diverse and active root biomass in the G treatment compared to the A soil (Hirsch et al., 2009). The results from the Highfield contrast with Jensen et al. (2017) who found that for the sandy loam soil at Askov, *E_sp_* of aggregates did not significantly differ between the UNF, ½ NPK, 1NPK and 1½AM treatments at field capacity (−100 hPa matric potential). Our results showed that geometric mean of *E_sp_* across the four aggregate size classes in air-dry state increased with increasing SOC content, although the relatioship was weak (Fig. 1b). In the wet state (−100 hPa matric potential), aggregates from the G treatment were stronger based on *E_sp_* than aggregates from the BF, A and LA treatments. Although *E_sp_* may include some plastic strain energy, the larger *E_sp_* for G implies that it is less susceptible to plastic deformation than the other treatments in a wet condition. Lower *E* was observed for the G aggregates at -300 and −1000 hPa matric potentials compared to the BF treatment. This can be interpreted as the G soil aggregates were more elastic than the BF soil. The influence of SOC on aggregate elasticity is further illustrated in Fig. 1c showing a strong negative linear decrease in *E* with increasing SOC content. Gregory et al. (2009) reported that compressed remolded soil cores from the G treatment were more elastic than the A treatment. Further, the authors found that the initial recovery of void ratio, used as an index of resilience after compression was greater in the G treatment (0.28–0.80) than the A treatment (0.16–0.58). This is an indication that the G soil cores were more elastic and rebounded more than the A soil cores following the removal of the compression stress.

### 4.2 Effect of soil organic carbon on water contents for tillage

The G and 1½AM soils with large SOC content had wider Δ*θ*_RANGE_ compared to their counterpart BF and UNF soils, respectively that had small SOC contents (Fig. 3a and b, Highfield soil; and Fig. 3c and d, Askov soil). The results support our hypothesis that increased SOC widens the range of water contents for tillage. Our results agreed with Munkholm et al. (2002) who determined Δ*θ*_RANGE_ using the consistency approach for soil from two of the experimental fields in Askov, which have the same sandy loam texture as the field investigated in the present study. The authors also reported that for both fields, Δ*θ*_RANGE_ was wider for the animal manure (AM) soil (0.09 kg kg^−1^) than the UNF soil (0.06 kg kg^−1^).The wider Δ*θ*_RANGE_ (consistency) for the G soil at Highfield (0.11 kg kg^−1^) compared to what was reported by Munkholm et al. (2002) can be explained by the differences in soil type, i.e., the silt loam soil at Highfield compared to sandy loam soil at Askov, as well as the wider range of SOC content for the Highfield soil compared to the Askov soil. The positive linear relation between SOC and Δ*θ*_RANGE_ showed that an increase in SOC content could potentially improves the window of opportunity for tillage operations by increasing Δ*θ*_RANGE_ over which tillage can be satisfactorily executed. Mosaddeghi et al. (2009) reported that SOC has greater absorptive capacity for water and improves water-holding capacity of soil thereby increasing *θ*_WTL_, *θ*_OPT_, *θ*_DTL_ and Δ*θ*_RANGE_. Moreover, SOC influences the plastic behavior of soil by shifting the plastic limit to greater water content (Kirchhof, 2006).

We observed that using the water retention approach, the *θ*_DTL_ was very dry, especially for the A treatment (0.08 kg kg^−1^), whereas it was very wet (wetter than −100 hPa matric potential) for the BF soil (Fig. 3a); which seems unrealistic. Similarly, we observed that *θ*_DTL_ estimated from the water retention approach was wetter than −100 hPa matric potential for all the treatments studied in Askov (Fig. 3c). Mueller et al. (2003) reported that *θ*_OPT_ estimated using the water retention approach was, generally, wetter than other approaches such as the consistency approach evaluated for 80 soils with differences in terms of geographical origin, parent material, texture, bulk density and SOC content. They found that *θ*_OPT_ was outside the suitable range of soil workability in the field. It must however, be emphasized that Mueller et al. (2003) only estimated *θ*_OPT_ using different approaches, but did not investigate *θ*_WTL_, *θ*_DTL_, and Δ*θ*_RANGE_ as done in this study. Dexter et al. (2005) and Dexter et al. (2008) suggested that although the water retention approach works for many soils, it does not work well for soils with bi-modal pore size distribution. This is because the van Genuchten equation assumes that soils have uni-modal pore size distribution. The pore size distribution calculated by numerical differentiation of the raw water retention data for the G treatment at Highfield, and the 1½AM treatment at Askov showed that the pore size distribution of the soils studied are better expressed with bi-modal water retention model, e.g., Double-exponential water retention equation (Dexter and Richard, 2009) than with uni-modal model such as the van Genuchten equation (data not shown). This helps explain the limitation of the water retention approach for estimating the water contents for tillage discussed previously. We suggest that the water retention approach is modified to take into account soils that cannot be fitted well with the van Genuchten equation.

The consistency approach, unlike the water retention approach seems to give a more reliable estimate of the water contents for tillage for the soils studied here by indicating when the soils were either too wet at *θ*_WTL_ or too dry at *θ*_DTL_ As for the consistency approach, *θ*_WTL_ was estimated from remolded soil (where air-dry soil sieved to 1 mm was remolded) destroying the soil structure and therefore, does not represent soils with intact structure. Moreover, plastic limit (PL) does not take into consideration pre-existing cracks which are important in soil fragmentation (Keller et al., 2007). There is a potential of using pedotransfer functions to estimate PL of soils. For example, Keller and Dexter (2012) proposed estimating PL from soil texture and clay content.

With respect to the determination of *θ*_DTL_, even though Dexter et al. (2005) provided a reasoning for defining *θ*_DTL_ as water content at which soil strength is twice its value at the *θ*_OPT_ as done in this study, they acknowledged that the approach provides an arbitrary way of determining *θ*_DTL_. We propose that a fixed value is defined for *θ*_DTL_. There is also a potential of using pedotransfer functions to estimate soil strength increases with decreasing water content to help reduce arbitrariness associated with the consistency approach.

### 4.3 Utilization of water contents for tillage and SOC information in farm management

Knowledge of the water contents (wet and dry limits) for tillage is useful for determining the range of water contents over which soil is workable, i.e., tillage can be performed satisfactorily. In temperate regions like Northern Europe, where soil workability is likely to be limited by excessive moisture, information on *θ*_WTL_ is of utmost importance to: (1) avoid producing soil seedbed dominated by large smeared fragments during tillage, which are of less agronomic value in terms of crop establishment (Dexter and Birkas, 2004); and (2) reduce the risk of soil puddling and remolding leading to excessive soil deformation and damage to the soil microstructure.

Knowledge of *θ*_DTL_ is also useful to: (1) avoid soil pulverization during tillage because seedbeds become dominated by both large intractable clods and very fine particles (dust) leading to poor aeration, vulnerability to crusting and greater erodibility (Braunack and Dexter, 1989); and (2) prevent the use of excessive tillage energy because soil is too strong. In these circumstances where clods are difficult to break down, considerable energy is expended to little or no effect. In a nutshell, quantitative information on the water contents for tillage can be used by farmers and environmental managers to improve their decision support system (DSS) for planning and optimizing tillage operations (Edwards et al., 2016).

Mullins et al. (1988) reported that in practice, farmers can be faced with a narrow window of opportunity to perform tillage operations, especially for hard-setting soils. Our results suggest that for the same soil type, increase in SOC increased the Δ*θ*_RANGE_. This information can provide practical evidence to farmers to engage in farm management practices that improve SOC as a way of widening the window of opportunity over which tillage can be performed satisfactorily.

It should be emphasized that for practical purposes before the application of our results in a DSS, it is important that the more promising consistency approach for determining the range of water contents for tillage, is validated under field conditions. Also, more knowledge is needed on the effect of SOC on different soil types and at different scales. It should also be pointed out that the high values of SOC associated with the G treatment may be due in part to the fact that it has not been cultivated. Cultivating it would lead to a sharp drop in SOC over time. However, the scope of this study could be expanded to identify appropriate conditions for grazing without risk of damage (poaching) to the underlying soil structure.

## 5 Conclusions

This study showed that the different long-term management practices on two contrasting soils lead to differences in soil organic carbon (SOC). This in turn led to major differences in soil mechanical properties (aggregate tensile strength, rupture energy and Young’s modulus and elastic range) which are useful in identifying appropriate soil moisture conditions for tillage. Two approaches were used to identify the range of soil water contents for tillage: (i) Based on fixed points (water contents) generated from modeled water retention characteristics and (ii) based on a combination of soil consistency relationships (plastic limit) and an estimate of tensile strength of aggregates in the 8–16 mm size class. The evidence here suggests:

• The aggregates from the Grass (G) treatment with large SOC content were stronger based on the mass specific rupture energy when soil was wet than the Bare fallow (BF) soil with small SOC content.
• Aggregate tensile strength for the G treatment was significantly lower than the Arable (A) and BF, and more elastic than the BF, A and Ley-arable (LA) treatments when soil was moist.
• The soil consistency approach provided more reliable estimates of tillage limits (upper, optimum and lower soil water contents) than the water retention approach.
• Management practices leading to increased SOC content can improve soil workability by increasing the range of soil water contents suitable for tillage (Δ*θ*_RANGE_) —SOC explains 78 and 87% of the variation in Δ*θ*_RANGE_ for the studied soils.

## Conflicts of interest

None.
